# Effects of different pretreatment methods on the drying characteristics and quality of potatoes

**DOI:** 10.1002/fsn3.1579

**Published:** 2020-09-29

**Authors:** Xiangfeng Sun, Xin Jin, Nan Fu, Xiaodong Chen

**Affiliations:** ^1^ Suzhou Key Laboratory of Green Chemical Engineering School of Chemical and Environment Engineering College of Chemistry, Chemical Engineering and Materials Science Soochow university Suzhou China

**Keywords:** blanching, citric acid, drying, moisture adsorption, potato, vitamin C

## Abstract

The effects of different pretreatments on the vitamin C content of peeled fresh potato, the drying characteristics, and several quality attributes of dehydrated potatoes were investigated. Citric acid pretreatment (0.1%–0.3%, 10–30 min), steam blanching (100ºC, 1–2 min), and water blanching (95°C, 1–2 min) were found to have no obvious effect on the drying rate of potatoes, whereas temperature was the main influencing factor. In terms of quality of dehydrated diced potato, 20 min of citric acid pretreatment resulted in the highest vitamin C retention and better color. Furthermore, dehydrated potato pretreated with citric acid all showed similar dynamic moisture adsorption curves, namely type II sorption isotherm. The moisture adsorption curves can be well fitted using the Guggenheim–Anderson–deBoer model with *R* ^2^ higher than .97.

## Graphical abstract

In this study, the 0.2% citric acid for 20 min was the best pretreatment method, which can made drying potato obtain the better color and high vitamin C.

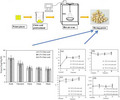

## 1 INTRODUCTION

Potato is one of the most popular foods and ranks the fourth among the five major crops (after wheat, rice, and corn, before soybean). Fresh potato is rich in nutrients including a variety of minerals, and its vitamin C content can reach to 42 mg/100 g among the five major crops (Burlingame, Mouillé, & Charrondière, 2009). Fresh‐cut fruits and vegetables are convenient and easy to prepare for consumers (Brecht, 1995; Luo & Barbosa‐Canovas, 1996; Saltreit, 1997). However, the peeled potato is very prone to browning and has a short storage time compared to the whole one. Therefore, it is essential to find efficient and simple approaches to extend the storage time and stabilize the color of potato after peeling.

Among the various pretreatment technologies for fresh‐cut produce, the most common ones are blanching, chemicals, freezing, ultrasound, and high pressure (Jambrak, Mason, Paniwnyk, & Lelas, 2007; Yu, Ma, & Bi, 2010). In particular, the addition of chemical inhibitors could suppress browning (Friedman, 1996). Sulfites can effectively control enzymatic browning (Sapers, 1993). However, alternatives are needed due to consumers’ health awareness and government bans on their use in fresh fruits and vegetables (Food & Drug Administration, 1989). Citric acid was found to inhibit the browning of fresh Chinese water chestnuts and extend the shelf life (Jiang, Pen, & Li, 2003). Researchers such as Goyeneche, Agüero, Roura, and Di Scala (2014) and Ducamp‐Collin, Ramarson, Lebrun, Self, and Reynes (2008) have shown that radish and lychee maintain their color after citric acid treatment. Besides, it was found that psychrophilic and mesophilic microorganisms on fruits and vegetables could be inhibited by citric acid (Bari et al., 2005; Uyttendaele, Neyts, Vanderswalmen, Notebaert, & Debevere, 2004). Researchers at home and abroad have extensively researched the optimum citric acid concentration and soaking time for different fruits and vegetables. However, only a few studies have considered it for pretreating potatoes (Calder, Kash, Davis‐Dentici, & Bushway, 2011; Xu, Li, & Zhao, 2015).

Water blanching and steam blanching have remained as popular commercial pretreatment methods, since they are easy to establish and implement. In general, water blanching is carried out at 80–100°C for 20 s to 20 min. Moisture sorption/desorption isotherms are essential for predicting stability during storage of foods, which influenced the shape and quality of foods.

In this paper, the effects of pretreatment with citric acid, steam blanching, and water blanching on the vitamin C content of fresh potato, the drying characteristics, and the quality and dynamic moisture adsorption characteristics of dried diced potato were investigated, in order to identify the best pretreatment technology. Considering food safety, the concentration of citric acid used during the experiments did not exceed 2% (McEvily, Iyengar, & Otwell, 1992).

## 2 MATERIALS AND METHODS

### 2.1 Materials

Fresh, whole, and bud‐free potatoes were purchased from the Hanlin Neighborhood Fresh Market in Suzhou Industrial Park, Jiangsu Province, China. The potatoes were peeled and cut into cuboids (1.2 × 1.2 × 1.1 cm) using a stainless steel knife.

### 2.2 Pretreatment of potatoes

The cut potato was blanched for a shorter time according to the size of the potato pieces. The following pretreatments were tested, using three samples in each one.

1. Water blanching (WB): Boiling in 50 ml water at 95°C for 1 or 2 min, then placing in crushed ice for 2 min to cool. The surface moisture was dried with a paper towel.
2. Steam blanching (SB): Treatment in a steam box (100°C) for 1 or 2 min, then placing in crushed ice for 2 min to cool. The surface moisture was dried with a paper towel.
3. Citric acid (CA) pretreatment: Soaking in 20 ml CA solution (0.1%, 0.2%, or 0.3% w/v) for 10, 20, or 30 min. Then, the samples were taken out and wiped with a paper towel.

### 2.3 Hot air drying experiments

The hot air drying experiments were carried out at air temperatures of 50, 60, and 70°C in a hot oven. Drying experiments were proceeding until sample weight became constant. When this condition was reached, there is a dynamic equilibrium between the sample equilibrium content moisture Me and drying air humidity. The sample moisture and solid contents were determined using the hot air at 150℃ for 24 h. Sample weight was recorded at an interval of 15min using an electronic balance (SATORIUS, BASA2202S, China) with a range of 2,200 g and system error of ±0.001g.

The drying kinetics was represented by the moisture ratio (MR, Equation 1) as a function of time:

(1) $$MR=\frac{M_{t}-M_{e}}{M_{0}-M_{e}}$$

where *M_t_* is the moisture content at time *t*, *M_0_* is the initial moisture content, and *M_e_* is the equilibrium moisture content.

### 2.4 D*etermination of vitamin C*

The ascorbic acid was analyzed according to Toor & Savage (2005) with modification. Sample with 2 g, extracted, was mixed with 25 ml oxalic acid dehydrate (OAD) (purity ≥ 99.5%., Sinopharm Chemical Reagent) solution (2 g/100 g OAD) by using a mechanical homogenizer. After 20 min at 10,000 rpm at 4°C, the supernatant was collected in a new tube. The vitamin C was assayed by using 2, 6‐dischlorophenolindophenol method. The results are expressed in milligrams of vitamin C per 100 g of fresh potatoes. The retention of vitamin C in fresh pretreated potatoes and dehydrated potatoes was calculated from the ratio of the measured vitamin C content to the fresh potato vitamin C content.

### 2.5 Determination of color

The color of the sample was measured with a CR‐100 colorimeter, and the samples were measured three times, and the color was expressed by the parameters L*, a*, and b* of the color difference meter. Value of lightness (L*) is 0 (black) to 100 (white); value of a* is −60 (green) to 60 (red); and value of b* is −60 (blue) to 60 (yellow). The total color difference was calculated by using Equation (2) (Nsonzi & Ramaswamy, 1998).

(2) $$\;\Delta E=\sqrt{{\left(L^{*}-L_{0}^{*}\right)}^{2}+{\left(a^{*}-a_{0}^{*}\right)}^{2}+{\left(b^{*}-b_{0}^{*}\right)}^{2}}$$

### 2.6 Dynamic moisture adsorption model

The Guggenheim–Anderson–deBoer (GAB) model mathematically describes the relationship between the relative humidity and the equilibrium moisture content. This model can fit a wide range of isothermal adsorption lines for foods. The corresponding equation is as follow:

(3) $$\frac{X}{X_{m}}=\frac{C_{g}\cdot K\cdot a_{w}}{\left(1-K\cdot a_{w}\right)\cdot \left(1-K\cdot a_{w}+C_{g}\cdot K\cdot a_{w}\right)}$$

where X is the moisture content [kg/kg on dry basis], X_m_ is the single‐layer number of molecules or single‐layer moisture content [kg/kg on dry basis], a_w_ is the water activity, C_g_ is the thermodynamic proportionality constant, and K is a constant.

### 2.7 Data processing and analysis

Statistical analysis was conducted using Microsoft office software. Significant differences (*p* < .05) between means were evaluated by one‐way ANOVA and Scheffer test. Graphics were drawn by Microsoft office software and origin 8.5.

## 3 RESULTS AND DISCUSSION

### 3.1 Effect of pretreatment on vitamin C content of fresh potato

Vitamin C is an essential nutrient for humans by preventing diseases like scurvy. Since it is water‐soluble, easily oxidized, and thermo‐sensitive, vitamin C in foods can be easily degraded depending on the processing and storage conditions (temperature, pH, light, time, and so on). Thus, it is often used as an indicator of the quality of fruits and vegetables (Goula & Adamopoulos, 2006).

**FIGURE 1:**
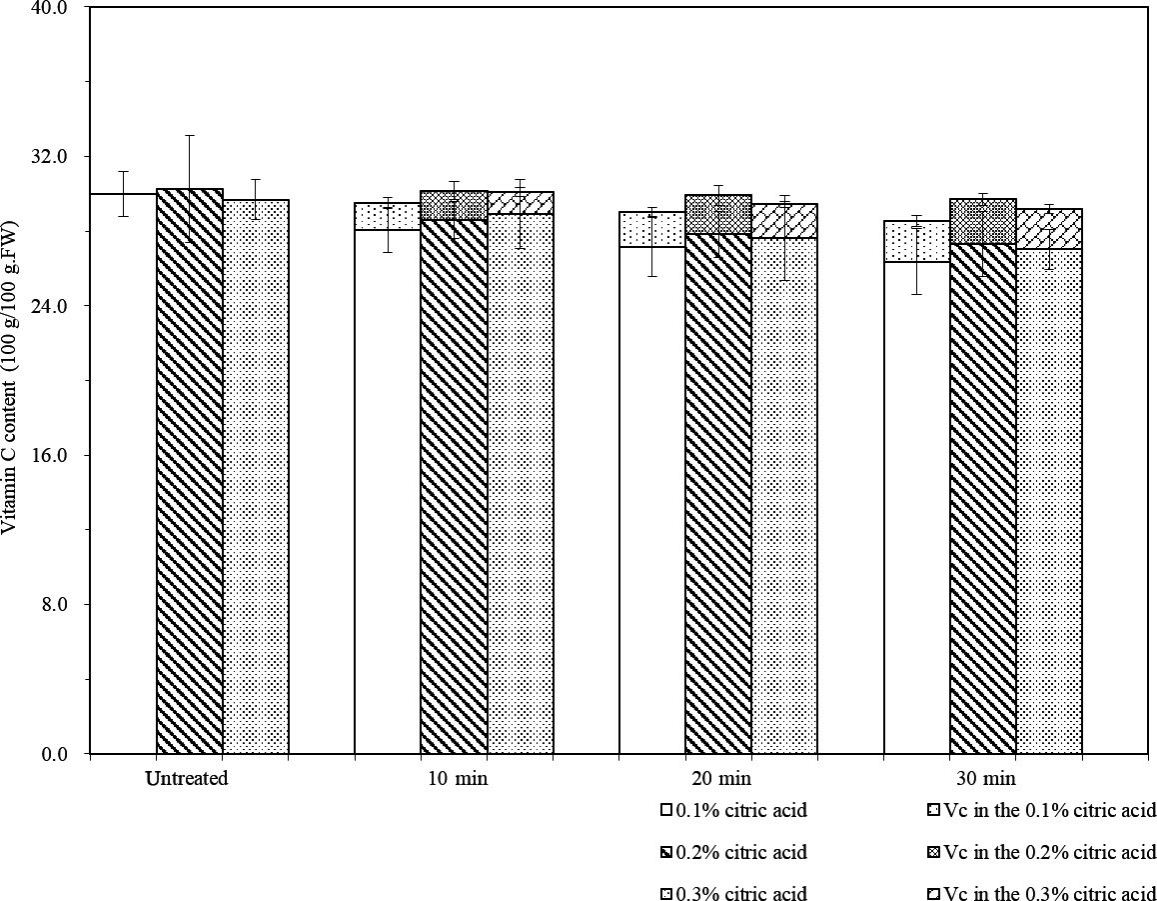
Effect of citric acid pretreatment on the vitamin C content of fresh potato Notice: Vitamin C in 0.1 ~ 0.3% citric acid solution represents content of vitamin C in citric acid after pretreated by 0.1 ~ 0.3% citric acid of fresh potato

Figure 1 shows the effect of different CA pretreatment conditions on the content of vitamin C in fresh potato. The initial content was 28.26–30.25 mg/100 g fresh weight, which is within the range reported by another group (2.8–42 mg/100 g fresh weight; Burlingame et al., 2009). The pretreatment time had no significant effect on the vitamin C content (*p* > .05). However, the CA solution after pretreatment contained a small amount of vitamin C (1–2.5 mg/100 g fresh weight), which also increased with the pretreatment time (*p* < .05). Therefore, a small amount of vitamin C must have been leached out of the potato during the pretreatment, although most of it was retained.

**FIGURE 2:**
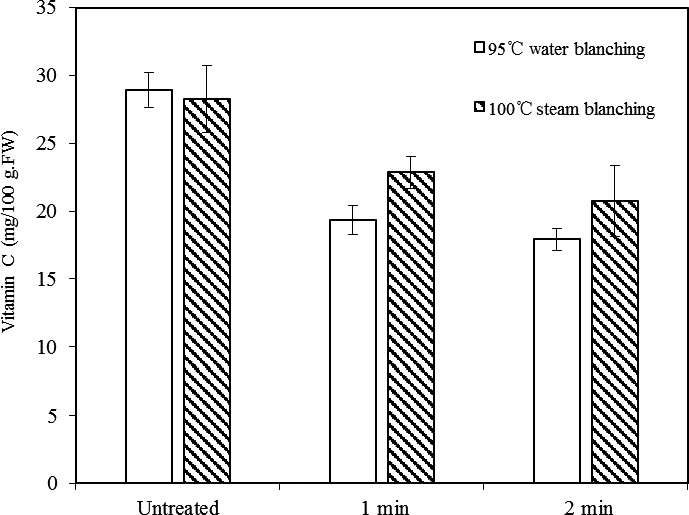
Influence of water and steam blanching on the vitamin C content of fresh potato

Figure 2 shows the effect of different blanching methods on the content of vitamin C in fresh potato. The retention of vitamin C was 67% and 62% after water blanching for 1 and 2 min, and 81% and 73% after steaming blanching for 1 and 2 min, respectively. As expected, prolonged blanching in either water or steam reduced the level of vitamin C. The reason is that heating destroyed the cell structure to release a large amount of vitamin C from the cells, a part of which was oxidized by oxygen. Compared to water blanching, steam blanching caused less vitamin C loss, since in this method, the potato was not in direct contact with bulk water.

### 3.2 Effect of drying temperature and pretreatment on potato drying characteristics

**FIGURE 3:**
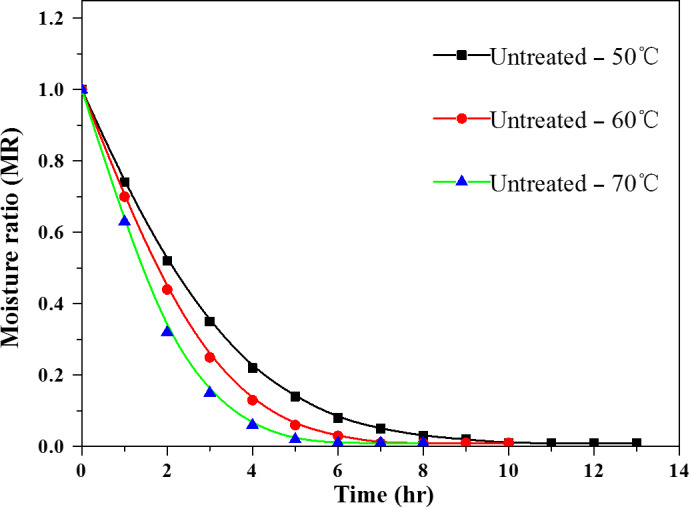
Effect of drying temperatures on the moisture ratio curve of potato cubes during drying

The effect of different drying temperatures on the drying characteristics of potato is shown in Figure 3. MR decreased considerably when increasing the drying time, similar to previous results for tomato (Demiray & Tulek, 2012), red pepper (Akpinar, Bicer, & Yildiz, 2003), and rosehip (Erenturk, Gulaboglu, & Gultekin, 2004). The equilibrium moisture content of the potato was measured after drying for 10, 8, and 6 hr at 50, 60, and 70°C.

**FIGURE 4:**
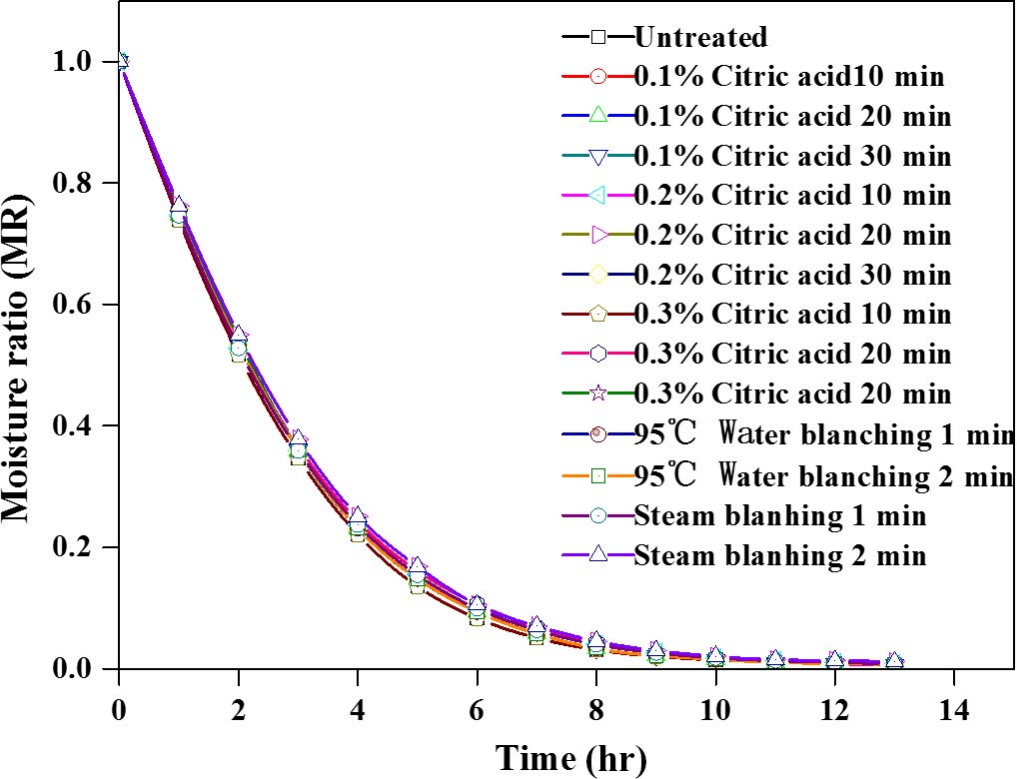
Effect of different pretreatment methods on moisture ratio curve of potato cubes during drying at 50°C

Figure 4 shows the effect of CA pretreatment, water blanching, and steam blanching on the drying of potato at 50℃. Similar drying characteristics were observed for all the pretreatment methods. On the other hand, the drying temperature was the main influencing factor.

### 3.3 Effect of different pretreatments on vitamin C content of dehydrated potato

**FIGURE 5:**
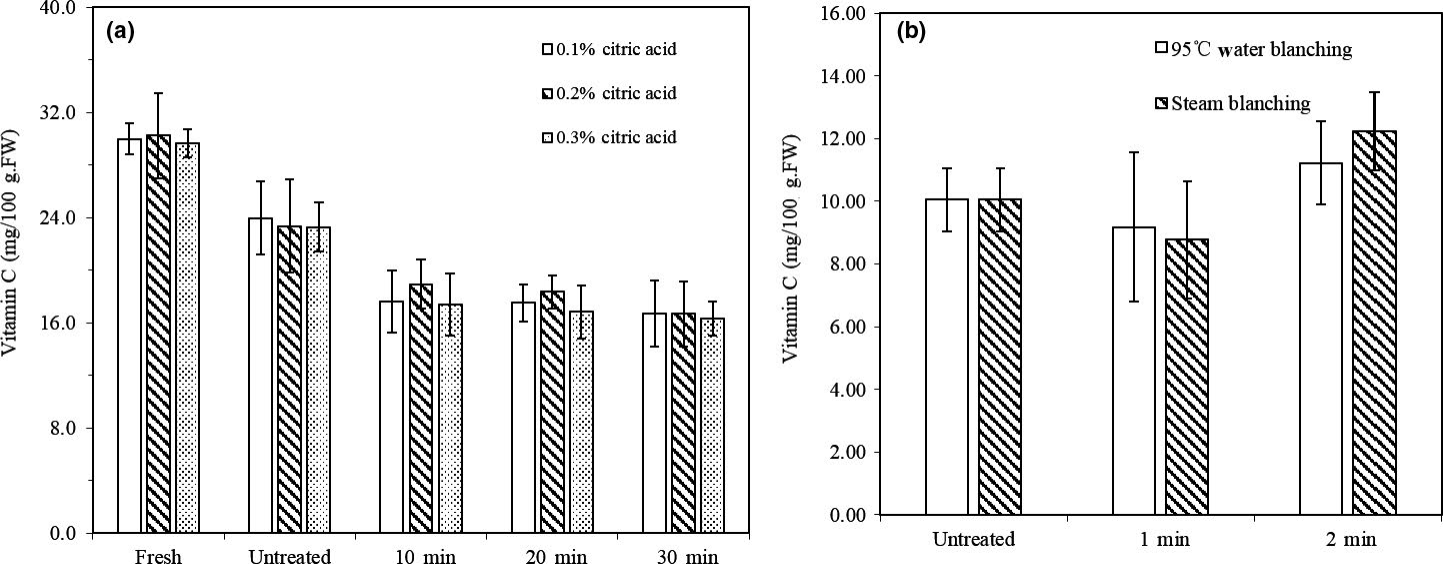
Comparison of the vitamin C content between dehydrated potato with different pretreatment methods and fresh potato. (a) Citric acid pretreatment, (b) water blanching at 95°C and steam blanching

Figure 5 shows the vitamin C content in the fresh and dehydrated potato samples. From Figure 5a, it can be found that the duration of CA pretreatment did not significantly influence the vitamin C retention in dehydrated potato (*p* > .05), which remained at 55%–61.6%. From Figure 5b, dehydrated water blanched potato showed the lowest vitamin C retention (44.1% and 38.3% for 1 and 2 min of blanching, respectively), due to severe leaching of vitamin C during the water blanching. The figure also shows that the retention of vitamin C in dehydrated potato was decreased when prolonging the blanching (*p* < .05). In addition, the untreated sample had the highest vitamin C content after dehydration (*p* < .05), because during CA treatment, a small amount of vitamin C was leached out, and the CA had no protective effect on the vitamin C during drying. Similar phenomena were reported for cashew apple and mangoes, in that the vitamin C content after dehydration became lower when the samples were first treated with an osmotic solution (Azoubel, El‐Aouar, & Tonon, 2009; Guiamba, Ahrné, & Khan, 2016).

### 3.4 Effect of different pretreatment methods on the color of dehydrated potato

Color is an essential parameter in food quality. The color of dehydrated vegetables may change significantly due to the Maillard reaction, pigment degradation, enzymatic browning, and oxidation of vitamin C during drying.

**FIGURE 6:**
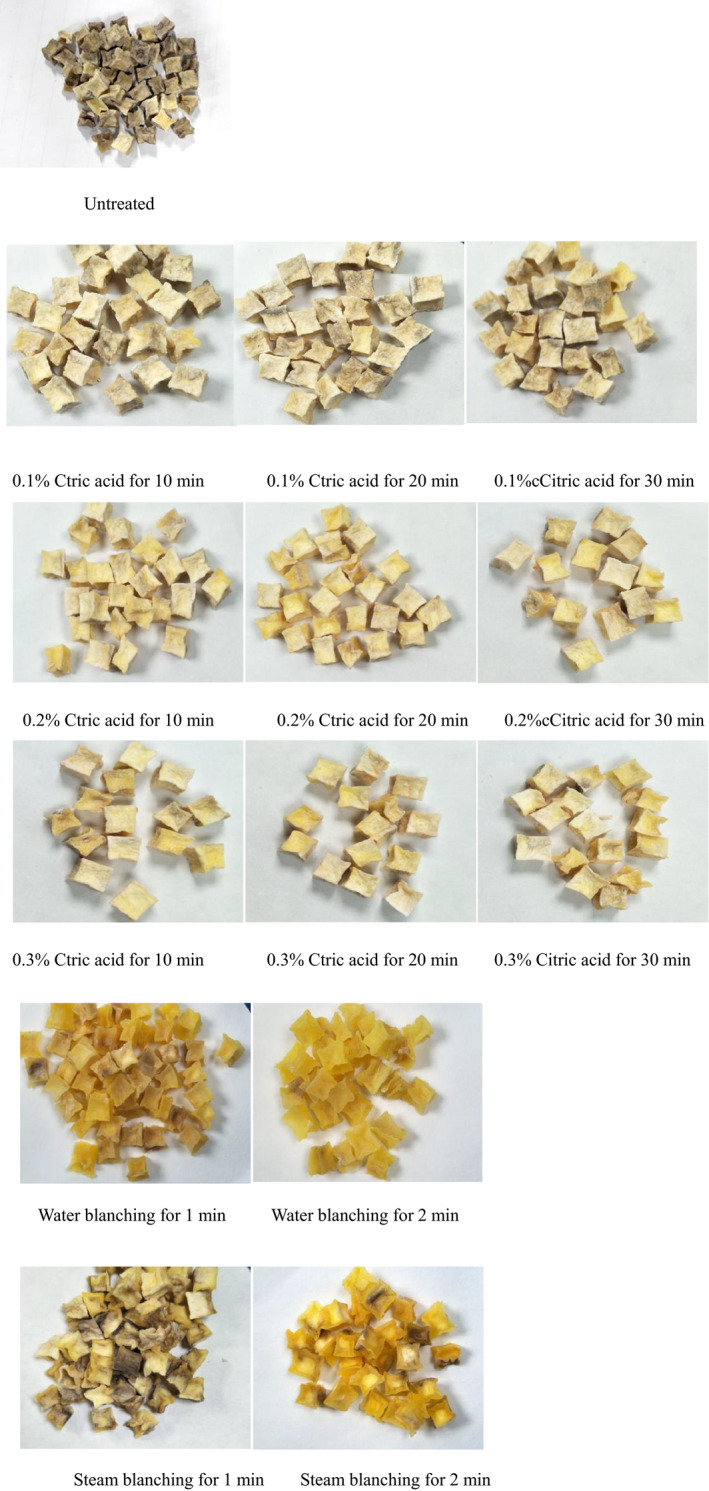
Color of different dehydrated potato cubes drying at 50°C

**FIGURE 7:**
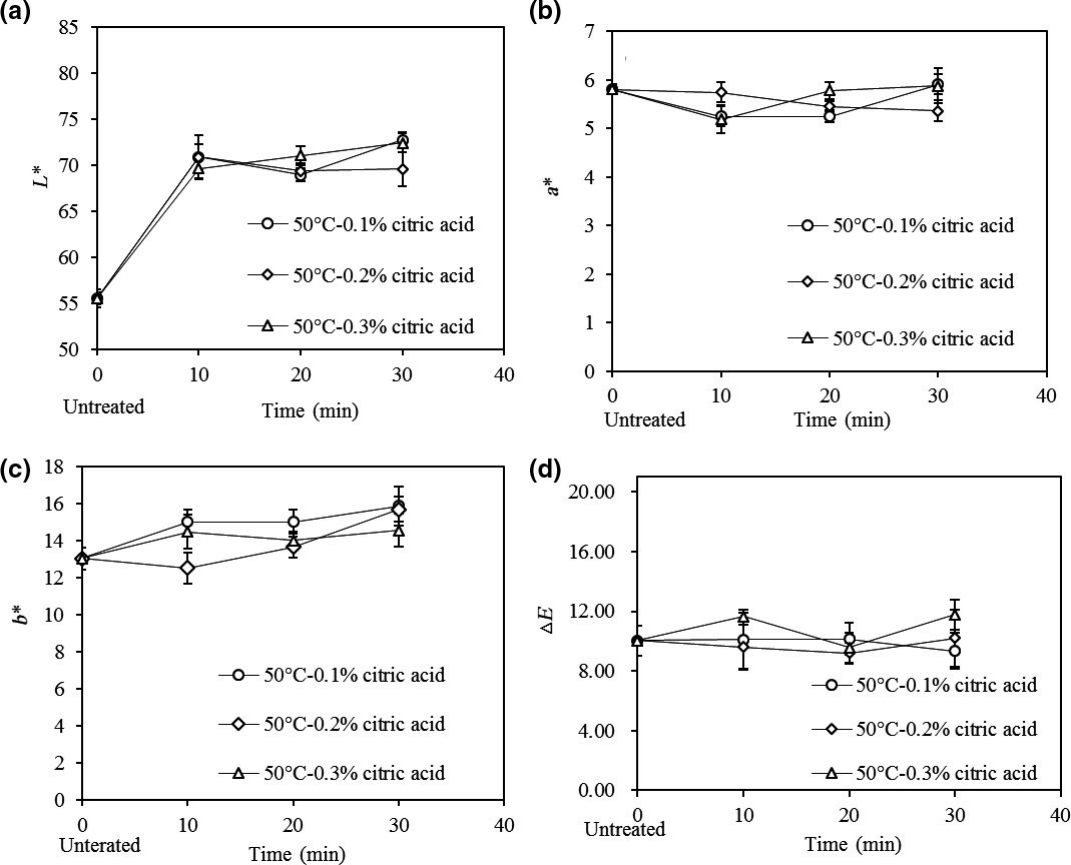
Changes in color parameters of dehydrated potato cubes subjected to citric acid pretreatment at different conditions followed by drying at 50°C. a: L* value, b: a* value, c: b* value, and d: ∆E value

**FIGURE 8:**
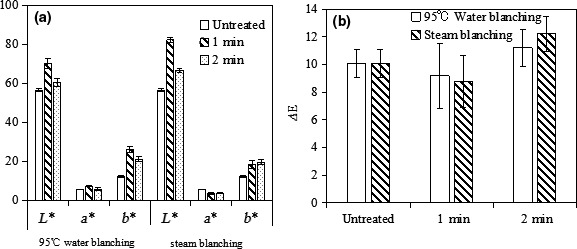
Changes in color parameters of dehydrated potato cubes subjected to water blanching and steam blanching followed by drying at 50°C. (a): L* value, a* value and b* value, (b): ∆E value

Figures 6, 7, 8 show the morphology and color value of dehydrated potato samples. In Figure 6, the untreated sample had the darkest color, which made it unappetizing. The samples pretreated with CA had the best coloration (light yellow) due to the color‐protecting effect of CA. Among the samples pretreated by blanching, those steam blanched for 1 min had the worst color. From Figures 6a and 7, the L* value (lightness) ranged from 55.56 to 72.77, with the lowest value (i.e., darkest) seen the untreated dehydrated potato. The duration of CA pretreatment did not influence the L* value. Meanwhile, a long blanching time increased the L* value, which possibly stems from stronger inhibition of polyphenol oxidase activity by blanching. From Figures 6b,c and 7, the values of a* (green–red) and b* (blue–yellow) were very similar for different dehydrated potato samples pretreated with CA (*p* > .05), whereas the water/steam blanching time had a significant influence on these values (*p* < .05). The a* and b* values of dehydrated potato range from 4.68 to 6.97 and from 12.51 to 27.3, respectively. The emergence of red color compared to the fresh potato is mainly due to the Maillard reaction during drying, and a burnt flavor is generated at the same time. High b* values (more yellow) were observed in dehydrated potato pretreated by water blanching for 1 min or by steam blanching for 2 min, at 23.52 and 27.3, respectively. Thus, these samples appear golden yellow in Figure 6. The color difference is a function of L*, a*, and b*. Thus, from Figure 8, it can be seen that the color difference of dehydrated potatoes was little (*p* > .05) due to the small difference of L*, a*, and b*. Considering all the experimental data, dehydrated potato pretreated in CA had better coloration, especially when the CA concentration was 0.2% for 20 min.

### 3.5 Effect of citric acid concentration on dynamic water adsorption characteristic of dried potato

**FIGURE 9:**
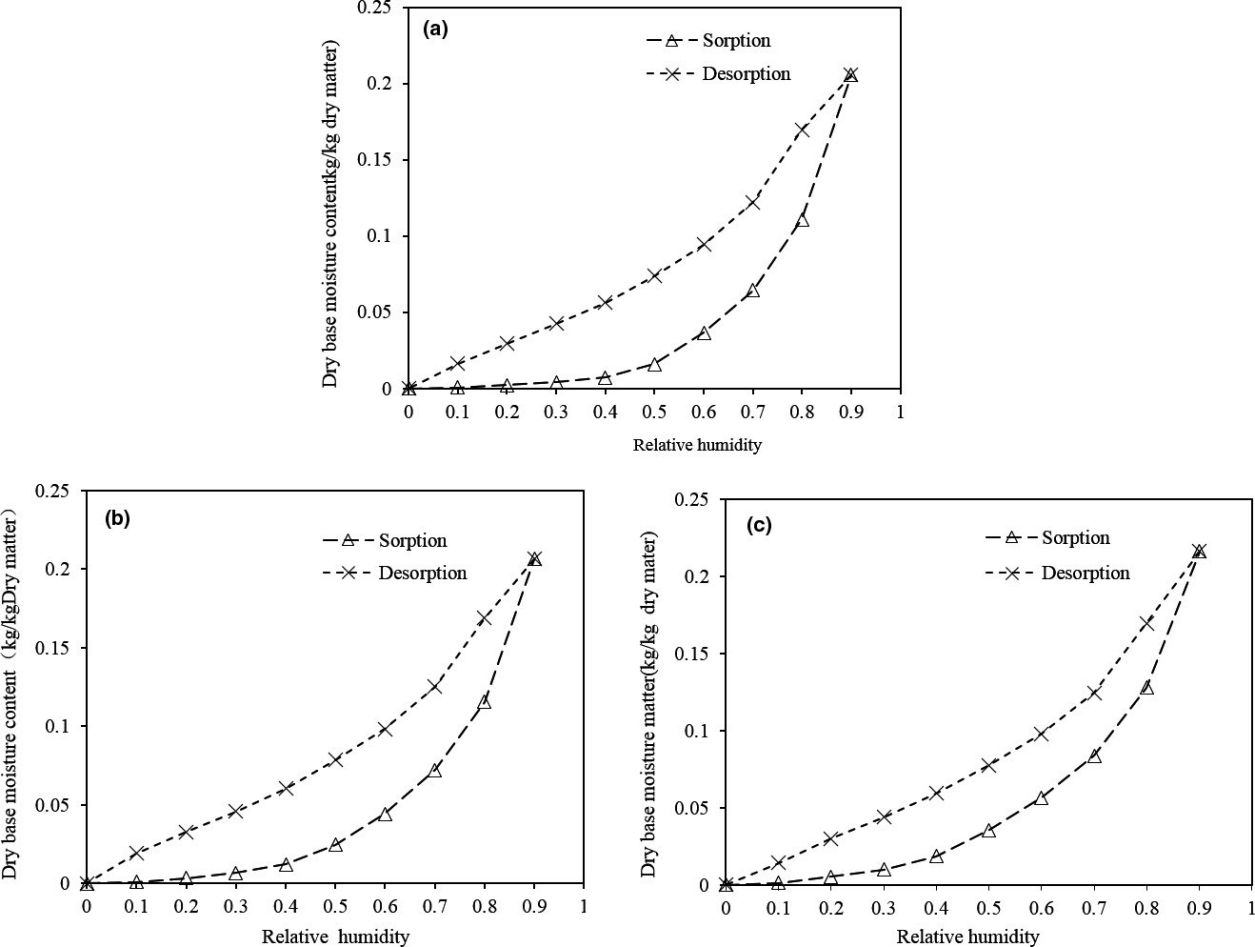
Adsorption and desorption curves of dehydrated potato cubes subjected to citric acid pretreatment at different conditions followed by drying at 50°C. a: 0.1% citric acid, b: 0.2% citric acid, and c: 0.3% citric acid

Figure 9a–c show the adsorption/desorption isotherms of potato pieces after pretreatment in CA solution at 50°C for 30 min using 0.1%–0.3% CA, respectively. The water adsorption was characterized by type II isotherm (Brunauer, Derming, Deming, & Troller, 1940). This shows adsorbing water of potato is from a gradual higher moisture uptake to small amounts of water uptake, as relative humidity increased. When dehumidifying, the moisture content on a dry base gradually decreased with decreasing relative humidity, and a relatively serious lag phenomenon occurred. This may mean that a part of the adsorbed water did not release during the desorption process due to interaction with certain nonaqueous components. And the concentration of CA has no obvious influence on dynamic moisture adsorption.

**FIGURE 10:**
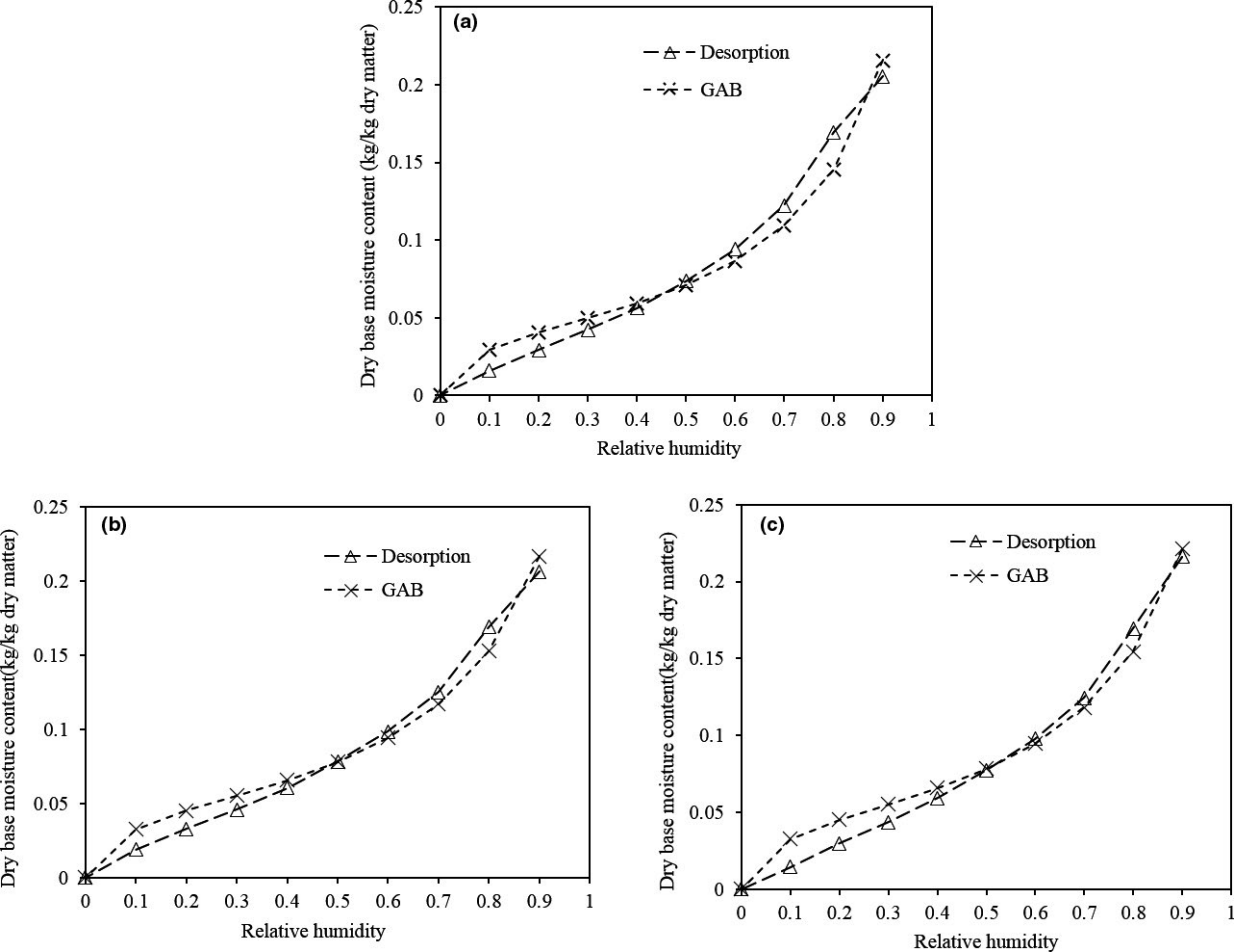
Comparison of desorption curves between experimental curves and GAB models for dehydrated potato cubes subjected to citric acid pretreatment followed by drying at 50°C. a: 0.1% citric acid, b: 0.2% citric acid, and c: 0.3% citric acid

The GAB model, based on Langmuir single‐molecule adsorption and Brunauer–Emmet–Teller (BET) multi‐molecular adsorption theory, mathematically describes the relationship between relative humidity and the equilibrium water content. This model can fit isothermal adsorption lines of various foods in a wide range. Figure 10 compares the measured desorption isotherm to that desorption isotherm fitted by the GAB model. In terms of the regression coefficient (*R* ^2^ > .97) and variance (S^2^) (Table 1), the GAB model has well goodness of fit, and therefore, it can be used to fit the desorption isotherm.

**Table 1:** GAB model parameters for dehydrated potato cubes pretreated by citric acid

Treated condition	*R* ^2^	*C* *_g_*	*X* *_m_*	S^2^
0.1% citric acid 30 min	.97091	18.00022	0.04176	0.001288
0.2% citric acid 30 min	.980372	18.000886	0.047081	0.000909
0.3% citric acid 30 min	.982847	18.0007	0.04702	0.001023

## 4 CONCLUSIONS

We investigated the effects of CA pretreatment, steam blanching, and water blanching under varying parameters on the vitamin C content of peeled fresh potato, the drying characteristics, and several quality attributes of the dehydrated potato. The vitamin C content did not significantly decline with CA pretreatment time, whereas the lowest content in fresh‐cut potato was 62% after blanching in water for 2 min. The drying temperature is the main factor influencing the moisture ratio. In the dehydrated potato, the best (i.e., lightest) color was observed in the sample pretreated with 0.2% CA for 20 min, which also had the highest vitamin C (61.6%). All dehydrated potato samples pretreated by CA showed similar dynamic moisture adsorption curves (type J curve and type II sorption isotherm), which could be well‐fitted by the GAB model with R^2^ values higher than 0.97. In summary, this study found that pretreatment in 0.2% CA solution for 20 min produced the best dried potato in terms of the moisture ratio, vitamin C, color, and dynamic moisture adsorption.

## 5 ETHICAL GUIDELINES

The authors declared that Ethics approval was not required for this research.

## CONFLICT OF INTEREST

The authors declared that they have no commercial or associative interest that represents a conflict of interest in connection with the work submitted.
